# Unpacking the cost of the lunchbox for Australian families: a secondary analysis

**DOI:** 10.1093/heapro/daad194

**Published:** 2024-01-10

**Authors:** Alexandra C Manson, Brittany J Johnson, Luke Wolfenden, Rachel Sutherland, Rebecca K Golley

**Affiliations:** Flinders University, College of Nursing and Health Sciences, Caring Futures Institute, Bedford Park, GPO Box 2100, Adelaide, SA 5001, Australia; Flinders University, College of Nursing and Health Sciences, Caring Futures Institute, Bedford Park, GPO Box 2100, Adelaide, SA 5001, Australia; Hunter New England Population Health, Hunter New England Local Health District, Wallsend, NSW 2287, Australia; School of Medicine and Public Health, University of Newcastle, Callaghan, NSW 2308, NSW, Australia; Population Health Research Program, Hunter Medical Research Institute, New Lambton Heights, NSW 2305, Australia; National Centre of Implementation Science, University of Newcastle, Callaghan, NSW 2308, Australia; Hunter New England Population Health, Hunter New England Local Health District, Wallsend, NSW 2287, Australia; School of Medicine and Public Health, University of Newcastle, Callaghan, NSW 2308, NSW, Australia; Population Health Research Program, Hunter Medical Research Institute, New Lambton Heights, NSW 2305, Australia; National Centre of Implementation Science, University of Newcastle, Callaghan, NSW 2308, Australia; Flinders University, College of Nursing and Health Sciences, Caring Futures Institute, Bedford Park, GPO Box 2100, Adelaide, SA 5001, Australia

**Keywords:** packed lunch, primary school, school food, nutrition, cost

## Abstract

Ninety per cent of Australian school children bring a home-packed lunch to school, with 44% of the food consumed during school hours being unhealthy. Among other factors, cost is a key consideration for food provision; however, the costs to Australian families are not well understood. Therefore, we aimed to determine what families are currently paying for school lunchboxes in Australian primary schools and to examine associations between food costs and socio-demographic factors with dietary quality. An audit of local retail outlets was used to determine the food costs of lunchbox contents. Costs (AUD) were adjusted for inflation as of early 2023. The lunchboxes of 1026 children aged 4–12 years at 12 Catholic primary schools in New South Wales, Australia, were assessed at the start of the day, using photography assessment methods and a validated School Food Checklist. The mean cost of lunchbox contents was $4.48 AUD (SD 1.53), containing a mean energy of 2699 kJ (SD 859), with 37.3% (SD 23.9) of energy sourced from unhealthy foods. Multiple linear regression analyses found that the strongest predictors of higher lunchbox cost (*P* < 0.05) were a higher proportion of energy from unhealthy foods (*B* = 0.016) and lower Socio-Economic Indexes for Areas (*B* = −0.178), when controlling for child socio-demographics. The results indicated that lunchbox food costs to Australian families are comparable to alternative school food service models in Australia and internationally. Results demonstrate the cost of food is not the only barrier to providing a healthy school lunchbox. Demonstrating a need for cost-considerate systematic interventions addressing food provision challenges and socio-economic disparities faced by families.

**Contribution to Health Promotion Statement**

- The school food environment is critical in health promotion, supporting food relationships, knowledge and dietary habits across the lifespan.
- Cost is an important consideration for Australian families, being identified by parents as a barrier in lunchbox food provision.
- Exploring demographics related to food knowledge, income and time for food preparation, which may be related to food costs can help inform tailored interventions to support populations.
- This study fills an evidence gap that is important to inform effective public health interventions to improve children’s diet quality in schools, demonstrating the potential of alternative provision models as a cost-comparable alternative.

## BACKGROUND

Childhood is a key stage for the development of healthy dietary habits. Dietary intake and food choices are established during childhood and adolescence, supporting growth and development (Mikkilä *et al.*, 2005). There are multifaceted influences on child health and development, including parent knowledge and beliefs, time and resource availability and socio-economic position (Anderson and Butcher, 2006; Rosenkranz and Dzewaltowski, 2008), making these factors important considerations for health promotion. Additionally, food insecurity is associated with developmental consequences and can contribute to reduced academic performance (Jyoti *et al.*, 2005).

School is a key health promotion setting, playing a critical role in the establishment of health and dietary intake habits during developmental years. Children internationally consume approximately one third of their daily energy intake during school hours, regardless of the food service model (Harrison *et al.*, 2013; Tugault-Lafleur *et al.*, 2017; Colombo *et al.*, 2020; Manson *et al.*, 2021). Hence, food consumed in school is important in dietary habit formation and an opportunity for health promotion. Food consumed by Australian children during school is typically sourced from home in a lunchbox, used by approximately 90% of students (Zarnowiecki *et al.*, 2018) or purchased from an onsite canteen/tuckshop. Other students may receive a meal from a lunch or food relief program; however, there is currently no universal safety net for food-insecure families or to address the dietary quality of children. Lunchboxes of primary school aged children are ordinarily packed by the parent/caregiver or student at home, to be consumed across the school break times (e.g. morning and midday eating breaks). However, Australian children’s school-time diets are currently profiled by a high intake of unhealthy foods (Sutherland *et al.*, 2020; Manson *et al.*, 2021), which are foods and beverages higher in energy, saturated fat, added sugars and/or sodium (National Health and Medical Research Council, 2013). This is consistent with the typical dietary quality of lunchboxes and packed lunches observed in the UK, Canada and the USA (Caruso & Cullen, 2015; Tugault-Lafleur *et al.*, 2017; Haney *et al.*, 2023). Further, the five food groups (National Health and Medical Research Council, 2013) are rarely consumed from lunchboxes during the school day, with vegetables, dairy and alternatives and meat and alternatives being consistently under-consumed in lunchbox systems internationally (Caruso and Cullen, 2015; Manson *et al.*, 2021; Haney *et al.*, 2023). As a key health promotion setting identified by the World Health Organization (World Health Organization, 2020), it is important to explore the factors related to school-time dietary quality to best support positive health and development outcomes and contribute to supporting lifelong health promoting dietary habits.

The cost of food is one of many considerations faced in lunchbox food purchasing and preparation by parents (Bathgate and Begley, 2011; Casado and Rundle-Thiele, 2015; Maher *et al.*, 2020; Watson-Mackie *et al.*, 2023). Literature investigating the perspectives of Australian parents when preparing lunchboxes has found food costs to be a commonly reported barrier, along with considering child preferences, limited time available for preparation, need for convenience and food safety concerns (Bathgate and Begley, 2011; Casado and Rundle-Thiele, 2015). Parent perceptions of these barriers have been discussed in relation to socio-demographic factors and family characteristics, including time availability, knowledge and disposable income availability (Harman and Cappellini, 2015; Hansen *et al.*, 2023; Watson-Mackie *et al.*, 2023). Barriers including cost may have varying influence on families across socio-demographics. To improve the dietary quality of lunchbox contents, the impact of food costs and the relationship between food costs and key demographic factors, including parent time availability, knowledge and socio-economic position, must be understood. This understanding could inform population-tailored public health strategies to alleviate the barriers to school food provision and improve the dietary quality of Australian children.

While the costs of canteen items in Australian schools have been described in previous research (Woods *et al.*, 2014; Wyse *et al.*, 2017; Billich *et al.*, 2019), little is known about the food costs of Australian school lunchbox provision. International studies have explored school-provided meal and lunchbox costs (Harper *et al.*, 2008; Caruso and Cullen, 2015; Wollny *et al.*, 2015; Bruckmayer *et al.*, 2021; School Nutrition Association, 2022), however Australian-specific studies are essential to reflect the unique food culture, school environment and food supply of this country. Therefore, the aims of this study are to determine what parents/caregivers are currently paying for food and beverage items in the lunchboxes of Australian primary school children and to examine associations between lunchbox food costs, socio-demographic factors and dietary quality.

## METHODS

### Study design

This study is a secondary analysis of cross-sectional data collected as part of a 2017 study in Newcastle, New South Wales (NSW), Australia (Sutherland *et al.*, 2020). Data collection and initial data preparation were completed by research staff at the Hunter New England Local Health District. The study was conducted according to the guidelines laid down in the Declaration of Helsinki and approval was obtained from the Hunter New England Human Research Ethics Committee (reference number 06/07/26/4.04), the University of Newcastle (reference number H-2008-0343) and the Maitland Newcastle Catholic Schools Office. This study is reported according to the requirements of the Strengthening the Reporting of Observational Studies in Epidemiology Statement for cross-sectional studies (von Elm *et al.*, 2007).

### Data source

#### Setting

Data were collected from February to March 2017, across 12 catholic primary schools located in the Hunter region of Newcastle, NSW (Sutherland *et al.*, 2020). The Hunter region includes major cities and regional areas. The region is characterized by a higher proportion of the population from low socio-economic backgrounds. These data were collected as the baseline assessment as part of a pilot study for the SWAP IT intervention to target food provided in the lunchboxes and physical activity of primary school children (Sutherland *et al.*, 2019).

#### Participants

Schools were eligible for inclusion in the study if they were a primary school, with over 120 enrolments and used the communication app ‘Skoolbag’, which has a large population reach and allowed for the SWAP IT intervention to be administered (Sutherland *et al.*, 2019). Only Catholic schools were included as the population for the broader factorial trial, with ethics approval granted by the Maitland Newcastle Catholic Schools Office (Sutherland *et al.*, 2019). Schools were excluded if they were participating in any other nutrition-based research studies, were a secondary school, or catered exclusively to children with special needs. School principals were contacted by Health Promotion Officers, who are employed within a population health service to support schools with health policies and programs, working collaboratively with research staff (Wolfenden *et al.*, 2017), via letter and follow-up phone call, with interested schools providing informed consent in a face-to-face meeting. Eligible schools were invited until 12 schools accepted the invitation to participate, with a sample size based on power requirements for the primary study (Sutherland *et al.*, 2019).

Parents/caregivers with a child at a participating primary school (kindergarten, which is the first year of primary school, to grade six) were invited to participate. Parents/caregivers were provided with a study information package and active parental consent was required. Children also provided assent to participate on the day of data collection.

#### Data collection

Data collection was completed by researchers from the University of Newcastle and the Hunter New England Local Health District. Child characteristics, including gender and school grade, were collected using the consent forms completed by parents/caregivers. Additionally, parental postcode, employment status and highest education qualification level were collected using a computer-assisted telephone interview with the consenting parents/caregivers.

Lunchboxes were observed at the beginning of a school day by trained research assistants. Child and parent/caregiver participants were not informed which day data collection would take place, to mitigate the influence of social desirability bias. At the beginning of the school day, prior to any food consumption, children were asked to display their lunchboxes on their desks and open any opaque containers or unidentifiable item packaging. Children who were sourcing lunch from the canteen on the day of data collection were excluded from the analysis. Eligible lunchboxes were labelled with a unique identification number during data collection. Children identified unclear items for researchers, who noted the items and photographed the lunchbox contents. Photographs were analysed by two trained dietitians to identify lunchbox items and reach a consensus decision, with a third assessor resolving any conflicts.

To support the identification and characterization of lunchbox contents, dietitians used the validated School Food Checklist (Kremer *et al.*, 2006; Mitchell *et al.*, 2010). This checklist includes 20 food and beverage categories and provides the average kilojoules per category and mean cost of items in Australian dollars (Kremer *et al.*, 2006; Mitchell *et al.*, 2010). Modifications to the School Food Checklist were made to enable the classification of items as ‘the five food groups’ (i.e. vegetables and legumes/beans, fruit, grain (cereal) foods, meat and alternatives, dairy and alternatives) or ‘unhealthy’ (i.e. discretionary food and beverages) according to the Australian Guide to Healthy Eating (National Health and Medical Research Council, 2013). The mean cost of food reflected the food prices as of 2017 when data collection occurred, using an audit of food and beverage prices in local retail outlets (Sutherland *et al.*, 2020) and costs assigned to each food item in proportion to grams. FoodWorks Professional Edition V7 (Xyris Software, Highgate Hill, QLD, Australia) was used as a guide for serving size and kilojoule per serving calculations (e.g. providing grams and kJ for one slice of bread) or via a database of nutrition profile of pre-packaged snack foods, developed by dietitians within the Hunter New England Population Health and University of Newcastle, based on an audit of Australian supermarket products.

### Secondary analysis

#### Data preparation

Socio-Economic Indexes for Areas (SEIFA) quintiles were identified using postcode of residence data according to 2016 Australian Bureau of Statistics data (Australian Bureau of Statistics, 2018). Percentage of energy sourced from the five food groups and unhealthy food was calculated as the proportion of total lunchbox kilojoules. Data were also grouped by those with 0%, 0.1–50% or 50.1–100% of energy from unhealthy food. This allows comparison between lunchboxes only providing serves of the five food groups, therefore meeting healthy lunchbox recommendations (NSW Government Health Department, 2023), against typical lunchboxes that contain unhealthy food and beverages (Manson *et al.*, 2021), and those that primarily consist of unhealthy food and beverages, which is not in line with dietary recommendations (National Health and Medical Research Council, 2013). Data checking and visual histogram assessment demonstrated that such grouping was appropriate, with a high frequency of healthy-only (0% energy from unhealthy foods) lunchboxes enabling distinct analysis of this group. Furthermore, the distribution of lunchboxes containing energy from unhealthy foods meant that the groupings accurately reflected the typical lunchbox profiles (median 34% IQR 23, 42) and not recommended lunchbox profiles (median 63% IQR 56, 78) from literature (National Health and Medical Research Council, 2013; Manson *et al.*, 2021). Food costs (AUD) were adjusted for inflation between early 2017 when initial costs were determined, and 2023 when the present analysis occurred, using Australia’s Consumer Price Index (CPI) for Food and Non-Alcoholic Beverage (Australian Bureau of Statistics, 2023b), with data sourced from the Australian Bureau of Statistics (Australian Bureau of Statistics, 2023a). This rise equated to an approximate 20% increase in CPI, from 106.0 in March 2017 to 127.6 in March 2023 (Australian Bureau of Statistics, 2023a). To reflect this, all lunchbox cost values were increased by 20.4%. Data were checked for normality, finding the data was normally distributed therefore mean and standard deviation are used in reporting.

#### Covariates

Regression analyses explored variables that can be incorporated into public health promotion strategies, to inform future programs. For example, demographics that could be considered in cost subsidies such as multiple children or socio-economic position, or known barriers to provision, such as time availability or knowledge to tailor interventions.

Socio-economic position was classified using the SEIFA Index of Relative Socio-Economic Disadvantage (2011). This index considers income, education and employment in specific living areas, therefore indicating the social and economic well-being in that region. Lower quintiles represent areas experiencing greater levels of disadvantage (Australian Bureau of Statistics, 2018). Parent/caregiver employment status was used as a proxy to reflect parent/caregiver time availability, as being time poor is commonly identified as being a major limitation to preparing lunchbox foods. This relates to the findings from Watson-Mackie *et al*., who found mothers who were not in the formal workforce had more time to prepare food that they considered healthy and avoid pre-packaged foods, in contrast with mothers working full-time (Watson-Mackie *et al.*, 2023). Additionally, international literature on both mothers and fathers demonstrates households with higher level of employment are more impacted by time barriers, contributing to convenience-based food choice coping strategies (Devine *et al.*, 2009; Wills *et al.*, 2011). Parent/caregiver education level was used as a proxy for parent/caregiver knowledge, with the highest level of education status being associated with higher nutrition knowledge (Hendrie *et al.*, 2008). Percentage of energy sourced from unhealthy foods was used to reflect the dietary quality of the lunchbox.

#### Potential confounders

Child characteristics including child gender and grade that have associated relationships with food intake in the literature but are not typically feasible to address in school interventions were treated as potential confounder variables.

#### Data analysis

Analyses were undertaken in IBM SPSS Statistics (Version 28; SPSS Inc.). Descriptive statistics were used to provide descriptive information on the mean and standard deviation for lunchbox food costs and food costs across a series of prespecified socio-demographic subgroups. Missing data and extreme outliers were excluded from the analysis and data checks were run to identify any errors.

Multivariate linear regression analyses were used to examine the relationship between child and family socio-demographic factors and lunchbox dietary quality with lunchbox food cost. Multivariate regressions included participants with complete socio-demographic and lunchbox data (*n* = 992). The model included family socio-demographic factors of interest and dietary quality, controlling for child characteristics. This included the independent variables of SEIFA, parent/caregiver education, parent/caregiver employment, number of children within the family enrolled in primary school and dietary quality of lunchbox, controlled for child gender and grade, and outcome of lunchbox food costs. The predictor variables were assessed for collinearity with no associations found. Parent/caregiver employment status categories were dummy-coded into new variables (Tabachnick and Fidell, 2013), with engaged-in-home duties coded as the reference category, based on hypothesized trends. SEIFA and education levels, which were ordinal categories, were coded numerically and treated as continuous variables in the regression (Tabachnick and Fidell, 2013; Pallant, 2020). All other variables were considered continuous for the analysis.

## RESULTS

One thousand and twenty-six children aged between 4 and 12 years consented to participate and completed data collection. Socio-demographic and anthropometric characteristics of the sample are presented in Table 1. The mean age was 7.9 years (SD 2.0). Participants were primarily distributed across SEIFA quintiles 1–4, aligned with the profile of the region, with 49.4% of responding parents/caregivers being university educated.

**Table 1:** Characteristics of the sample of Australian primary school children and their families (*n* = 1026)

	*N*	%
Child characteristics		
Age (years) (mean, SD)	7.9	2.0
Year level (mean, SD)	2.8	2.0
Gender		
Male/boys	538	52.4
Family characteristics		
SEIFA		
Quintile 1 (highest level of disadvantage)	221	21.5
Quintile 2	345	33.6
Quintile 3	260	25.3
Quintile 4	189	18.4
Quintile 5 (lowest level of disadvantage)	11	1.1
Parent/caregiver employment^a^		
Unemployed, student, other	39	3.9
Engaged in home duties	110	11.1
Employed part-time	467	47.0
Employed full-time	378	38.0
Parent/caregiver education level^b^		
Did not complete high school	66	6.7
Completed high school	102	10.3
TAFE Certificate or Diploma	334	33.7
University degree or other related	490	49.4
No. of children in primary school		
* One*	524	51.1
* Two*	409	39.9
* Three or more*	93	9.1
Remoteness		
Regional/remote Australia	178	17.3
Major cities	848	82.7

^a^Reported by one parent/caregiver. Thirty-two not reported/missing.

^b^Reported by one parent/caregiver. Thirty-four not reported/missing.

SEIFA, Index of Relative Socio-economic Advantage and Disadvantage (Australian Bureau of Statistics, 2018).

### Descriptive analysis of lunchbox cost

Table 2 presents children’s mean school lunchbox food cost, energy and dietary quality. The mean daily cost of a lunchbox was $4.48 AUD (SD 1.53) per child, containing a mean of 2700kJ (SD 859). Of the total energy, a mean of 37.3% (SD 23.9) was sourced from unhealthy foods.

**Table 2:** Descriptive results of the cost and dietary quality of lunchboxes of Australian primary school children (*n* = 1026)

	Mean	SD
Cost AUD$	4.48	1.53
Total energy kJ	2699	859
kJ from the five food groups	1607	611
kJ from unhealthy food	1092	864
Percentage of energy from unhealthy foods (%)	37.3	23.9

### Lunchbox cost across socio-demographic groups and dietary quality

Mean lunchbox food costs within socio-demographic groups are presented in Table 3. Children in Kindergarten and grade six had the highest lunchbox costs; however, there are no observable trends in the cost of lunchboxes with child grade. Male children had higher food costs for lunchboxes ($5.54 [SD 1.55]) compared to female children ($4.42 [SD 1.51]). Children with 100% of food sourced from the five food groups (healthy items only and aligned with recommendations) had lower cost lunchboxes ($3.62 [SD 1.18]) in comparison to those with mostly food from the five food groups, the typical lunchbox profile (<50% unhealthy foods) ($4.37 [SD 1.24]), and mostly unhealthy food, not aligned with recommendations (>50% unhealthy foods) ($5.15 [SD 1.96]). Children living in areas of most disadvantage had higher lunchbox costs (SEIFA quintile 1 mean = 4.64 [SD 1.88], quintile 2 = 4.68 [SD 1.49], quintile 3 = 4.49 [SD 1.37], quintile 4 = 3.99 [SD 1.27], quintile 5 = 3.65 [SD 0.70]). Parents/caregivers engaged in home duties had higher mean food costs ($4.59 [SD 1.92]) compared to other employment statuses. Mean cost differed across parent/caregiver education, with lower lunchbox cost for parents/caregivers with technical and further education ($4.48 [SD 1.58]) or University ($4.32 [SD 1.43]) level education compared with those who did/did not complete high school ($4.77 [SD 1.75], $4.79 [SD 1.52], respectively). Mean cost for families with one child in primary school was higher ($4.53 [SD 1.51]) compared to those with two ($4.43 [SD 1.55]) or three children in primary school ($4.46 [SD 1.59]).

**Table 3:** Mean school lunchbox food costs of Australian primary school children by child and family socio-demographic groups and dietary quality (*n* = 1026)

	*N*	Mean cost $AUD	SD
Child characteristics			
Grade			
Kindergarten	177	4.57	1.52
1	149	4.44	1.53
2	138	4.54	1.50
3	149	4.38	1.62
4	152	4.45	1.48
5	140	4.33	1.50
6	121	4.69	1.60
Gender			
Male/boys	538	5.54	1.55
Female/girls	488	4.42	1.51
Percentage of energy sourced from unhealthy foods			
Five food group items only	138	3.62	1.18
<50% energy sourced from unhealthy foods	616	4.37	1.24
>50% energy sourced from unhealthy foods	272	5.15	1.96
Family characteristics			
SEIFA quintiles			
1 (most disadvantaged)	221	4.64	1.88
2	345	4.68	1.49
3	260	4.49	1.37
4	189	3.99	1.27
5 (least disadvantaged)	11	3.65	0.70
Parent/caregiver employment^a^			
Unemployed, student, other	39	4.31	1.21
Engaged in home duties	110	4.59	1.92
Employed part-time	467	4.44	1.43
Employed full-time	378	4.45	1.53
Parent/caregiver education level^b^			
Did not complete high school	66	4.77	1.75
Completed high school	102	4.79	1.52
TAFE certificate or diploma	334	4.48	1.58
University degree or other related	490	4.32	1.43
Number of children in primary school			
1	524	4.53	1.51
2	409	4.43	1.55
3+	93	4.46	1.59

^a^Reported by one parent/caregiver. Thirty-two not reported/missing.

^b^Reported by one parent/caregiver. Thirty-four not reported/missing.

SEIFA, Index of Relative Socio-economic Advantage and Disadvantage (Australian Bureau of Statistics, 2018).

### Associations between socio-demographic factors and dietary quality with lunchbox cost

Multivariate linear regressions showed significant associations (*P* < 0.05) between proportion of energy from unhealthy foods (*B* = 0.016 [SE 0.002], *P* < 0.001), SEIFA Index (*B* = −0.178 [SE 0.045], *P* < 0.001), and cost of lunchbox contents, when adjusting for child gender and grade (*F* = 9,892 = 11.05, *P* = 0.001). There were no significant associations (*P* > 0.05) between parent/caregiver education level (*B* = −0.105 [SE 0.054], *P* = 0.052), number of children within the family enrolled in primary school (*B* = −0.118 [SE 0.072], *P* = 0.104) and parent/caregiver employment status (Unemployed *B* = −0.145 [SE 0.246], *P* = 0.556; Employed part-time *B* = 0.017 [SE 0.102], *P* = 0.870; Employed full-time *B* = 0.026 [SE 0.158], *P* = 0.867) and cost of lunchbox contents (Table 4).

**Table 4:** Associations of individual and family socio-demographics of Australian primary school children with the food costs of school lunchboxes

Variable	*B*	*SE B*	Standardized β	*P*
Proportion of energy from unhealthy foods	0.016	0.002	0.247	<0.001
SEIFA quintiles^a^	−0.178	0.045	−0.123	<0.001
Parent/caregiver education level	−0.105	0.054	−0.061	0.052
Parent/caregiver employment status vs. engaged in home duties				
Unemployed, student, other	−0.145	0.246	−0.018	0.556
Employed part-time	0.017	0.102	0.005	0.870
Employed full-time	0.026	0.158	0.005	0.867
Number of children within the family enrolled in primary school	−0.118	0.072	−0.050	0.104

Model controlled for gender, school grade and the number of children within the family enrolled in primary school.

*F* = (9,982) = 11.05, *p* = 0.001. *R* = 0.303, *R*^2^ = 0.092.

^a^Socio-economic index For Areas—Index of Relative Socio-Economic Disadvantage—2016—SA1—Quintiles.

## DISCUSSION

This study explored what parents/caregivers are paying for school food in Australia and the associations between lunchbox food costs with dietary quality and socio-economic position, using a sample of Australian primary school children. Lunchbox food costs were approximately $4.50 per child per day, with higher cost lunchboxes associated with poorer dietary quality of lunchboxes and higher level of disadvantage. The cost is comparable to alternative school food provision options, such as school-provided meals or canteens, in Australia and internationally (Caruso and Cullen, 2015; Delaney *et al.*, 2017; Smith, 2020; School Nutrition Association, 2022; New Zealand Ministry of Education, 2023). These results inform the key factors that are associated with higher lunchbox food costs to inform future public health strategies.

Lunchboxes containing higher proportions of unhealthy foods had higher food costs, indicating healthy food provision consisting of five food groups can be more affordable than the provision of unhealthy foods for the lunchbox. This finding contrasts with pricing in Australian school canteens, where healthy lunch items and snacks including salads, sandwiches and fruit, categorized as ‘green’ foods according to canteen guidelines are typically more expensive than the less healthy ‘red’ options, including pies, hot dogs and chips (Woods *et al.*, 2014; Wyse *et al.*, 2017; Billich *et al.*, 2019), likely in part due to the labour costs of preparing items (Jabs and Devine, 2006; Wyse *et al.*, 2017; Gaddis, 2019). The purchasing of lunchbox items from the supermarket is aligned with analyses of Australian habitual food budgets, with a recent analysis finding that food purchases according to Australian dietary guidelines are more affordable by $124 to $227 per fortnight than the current habitual diet, which contains >50% unhealthy foods, for all socio-economic groups (Lewis *et al.*, 2021). Furthermore, a costing of healthy and sustainable food baskets (median $188.21) compared with a typical Australian food basket ($224.36) found the healthy and sustainable basket was significantly (*P* < 0.05) less expensive and more affordable in all metropolitan areas and socio-economic quintiles (Goulding *et al.*, 2020). However, the authors noted it was important to consider the acceptability of the healthy and sustainable basket, which requires more preparation and cooking, demanding a higher skill level and time dedication (Goulding *et al.*, 2020). Qualitative interviews with Australian caregivers described the experience of packing lunchboxes, finding that parents/caregivers perceive healthy food as having higher costs and being inconvenient, therefore unhealthy and convenient foods are commonly provided in place (Bathgate and Begley, 2011). This perception may be due to the labour of healthy food provision (Jabs and Devine, 2006), including the burden of creating meals from affordable five food group ingredients; preparation, knowledge and time availability, while considering child preferences and palatability. This contrasts with commonly available ready-to-eat unhealthy lunchbox product items, which are convenient, food safe, require minimal labour and are acceptable to children (Jabs and Devine, 2006; Bathgate and Begley, 2011; Rathi *et al.*, 2018). Furthermore, many of these products feature child- or parent-directed marketing (Watson *et al.*, 2023). As a result, there is potential for the promotion of healthy lunchbox provision to reduce food costs, concurrently to increasing the availability and access to healthy, convenient and affordable food, to address the challenges faced by families.

Inequities exist in the food costs of lunchboxes, with the lower socio-economic position being associated with higher lunchbox costs, with a difference of up to $0.90 between the most and least disadvantaged quintiles, irrespective of unhealthy food content. These findings are consistent with limited previous evidence. Sanigorski and colleagues found lunches of children in the lowest socio-economic quartile in Victoria, Australia in 2003–04 were more expensive than those in the highest socio-economic quartile, with a $0.26 significant difference between groups (Sanigorski *et al.*, 2005). Additionally, results of a US study found that students attending lower income schools had higher cost home lunches than middle-income schools (Caruso and Cullen, 2015). It is likely there is a multitude of complex factors, which contribute to this discrepancy. These findings could be due to a greater total quantity of food provided and an increased proportion of costly convenient foods in the lunchboxes. Food access and availability challenges may influence food provision, with less availability of healthy or affordable options in areas of a higher level of disadvantage. This was found in a Sydney, NSW food basket survey, finding greater variety and quality of fresh fruit and vegetables in suburbs of a high socio-economic position, compared to suburbs of a low socio-economic position (Crawford *et al.*, 2017). Further, while nutritional and financial literacy may play a role in the provision of higher cost items, it is important to consider the varying impact of food provision challenges across different socio-economic groups, which were unable to be controlled for in the present study. Specifically, a systematic review of 28 international studies found socio-economic position was associated with predictors of dietary intake including home-environment factors, parent modelling and child nutrition knowledge (Zarnowiecki *et al.*, 2014). Furthermore, barriers to food provision influence families differently based on their situation, for example, a qualitative study with Australian mothers found that mothers who lacked time for preparation were less concerned about financial constraints, thus spending more on food (Watson-Mackie *et al.*, 2023). While parent/caregiver employment status, used as a proxy for time availability, was not significantly associated with cost when other demographics were controlled in the present study, the example from Watson-Mackie *et al*. demonstrates that there are many complex influences that can contribute to trade-offs in prioritization of food costs. Therefore, the impact of higher cost lunchboxes for families of low socio-economic position should be considered, as a larger proportion of income being spent to provide school food, reducing lunchbox affordability. The complexities of this relationship demonstrate the need for tailored, socio-demographic conscious programs aiming to address the unique barriers faced by families, to support good dietary quality in school.

The food costs of $4.48 per child per day lunchbox provision for parents/caregivers can be interpreted in the context of other school food provision models. Purchasing food for recess and lunch from an Australian school canteen can provide a relatively comparable alternative, with unpublished baseline data from a canteen intervention in NSW primary schools finding canteen lunch orders cost a mean of $4.69 (SD 1.80) (Delaney *et al.*, 2017). In Tasmania, Australia, a pilot trial in 2020 of a school-provided lunch program across three schools found that total costs for the lunches were an average of $4.72 per child per day for a nutritious single-option menu, including $1.91 for ingredients and $2.81 for labour (Smith, 2020). In New Zealand, the 2023 Ka Ora, Ka Ako Healthy School Lunches Programme was costed at a maximum of NZD $5.39 ($4.99 AUD) per child in grades 0–3 and $6.31 NZD ($5.84 AUD) for a larger portion for children in grades 4–8 for lunch each day (New Zealand Ministry of Education, 2023). Major costs for this program were described as food, preparation, delivery and staffing. The costs to families of the US government-subsidized school food model were reported by the School Nutrition Association. The average cost of school meals to families was USD $2.48, $2.68 and $2.74 daily ($3.35, $3.62, $3.70 AUD) to elementary, middle and high school students, respectively (School Nutrition Association, 2022). Additionally, a 2011 US study compared the mean price of home-packed lunches and the National School Lunch Program (Caruso and Cullen, 2015), finding school meal costs to families were USD $1.82 and $2.05 ($2.46, $2.77 AUD) for elementary and intermediate school students respectively, while the food costs for home-packed lunches were $1.93 and $1.76 ($2.61, $2.38 AUD). School-provided meal systems alleviate labour demands on families and can address parent/caregiver-identified barriers to lunchbox provision (O’Rourke *et al.*, 2020), increasing access to food for all school children. While limited research has explored parent/caregiver willingness to pay for a school-provided meal or healthy canteen provision in Australia, pilot data indicate families may be willing to contribute between $3 and 5 per child per day (Smith, 2020; Manson *et al.*, 2022). Therefore, the current costs of lunchboxes in the broader school food context demonstrate the potential feasibility of a cost-comparable, lower-burden alternative for Australian families.

### Strengths and limitations

This study provides comprehensive and contemporary insight into the food cost of Australian primary school children’s lunchboxes. A strength was the inclusion of evidence-informed covariates in the regression to ensure results were an accurate depiction of independent relationships and the large sample of primary school children. A key limitation was the single inflation rate applied to all products when adjusting costs to 2023, which may not consider greater inflation rates for certain food products, including differing cost changes for healthy products. Furthermore, as data were collected in 2017 it may not reflect recent changes as a result of rising costs of living in Australia and the discussed 20% inflation of food costs. These cost changes may have contributed to families adopting different purchasing patterns and led to varied lunchbox composition, which is not captured in the results. Additionally, the mean cost of food does not capture the influence of product discounts on purchasing trends or total lunchbox costs. While lunchbox contents were systematically assessed by trained dietitians, items were not weighed to determine exact quantities. Finally, despite the large sample, only including participants in one region of NSW attending catholic schools are captured, which may reflect families of an above-average household income due to increased Catholic school fees (Daniels, 2011), limiting the generalisability of the findings.

### Implications

Understanding the factors related to school food costs can inform policy and practice across the food system. Australian state and territory policies encourage the promotion of good dietary intake in the school community. To support this, future programs should aim to improve the access to healthy and affordable lunchbox foods, which have a low preparation burden for families, across all socio-economic groups. An avenue to achieve this may include increasing the availability and promotion of affordable, enjoyable, convenient and healthy foods in local supermarkets and school canteens. Internationally, school food systems should strive to consider how cost influences the choices of families to ensure there is equitable access to food in schools.

Future research should explore labour costs of school lunchbox preparation for families to understand the true cost and potential trade-offs made between labour and food costs. Further, research should explore the potential of improved lunchbox programs, school canteen offerings or a school-provided meal option in Australia to alleviate the labour burden for families, including time and convenience, at a low cost. Specifically, exploring parent/caregiver interest in a school meal system and the financial cost families are willing to contribute, including how contributions may differ across family socio-demographics or depending on what the system provides for families. Furthermore, food costs should be considered in the context of the household budget and experience of food insecurity, to determine school food affordability.

## CONCLUSION

The present study provides the most comprehensive and contemporary insight into the food costs of the lunchboxes of Australian primary school children for families. The food cost for lunchboxes of Australian primary school children is approximately $4.50 AUD, with higher cost for lunchboxes containing a higher proportion of unhealthy foods and families living in greater areas of disadvantage. These findings reiterate that the cost of healthy food is not the key barrier to providing a nutritious school lunchbox. Therefore, there is a need for cost-considerate systematic interventions that capture the multitude of school food challenges faced by families and the associated socio-economic disparities.

## Data Availability

The datasets used and/or analysed during the current study are available from the corresponding author on reasonable request.
